# Molecular characterization of chicken DA systems reveals that the avian personality gene, *DRD4*, is expressed in the mitral cells of the olfactory bulb

**DOI:** 10.3389/fnana.2025.1531200

**Published:** 2025-01-15

**Authors:** Toshiyuki Fujita, Naoya Aoki, Chihiro Mori, Koichi J. Homma, Shinji Yamaguchi

**Affiliations:** ^1^Department of Biological Sciences, Faculty of Pharmaceutical Sciences, Teikyo University, Tokyo, Japan; ^2^Department of Molecular Biology, Faculty of Pharmaceutical Sciences, Teikyo University, Tokyo, Japan

**Keywords:** personality gene, dopamine receptor, dopamine neuron, DRD4, chick, dopamine, olfactory bulb, mitral cell

## Abstract

Animal personalities are stable, context-dependent behavioral differences. Associations between the personality of birds and polymorphisms in the dopamine receptor D4 (DRD4) gene have been repeatedly observed. In mammals, our understanding of the role of the dopamine (DA) system in higher cognitive functions and psychiatric disorders is improving, and we are beginning to understand the relationship between the neural circuits modulating the DA system and personality traits. However, to understand the phylogenetic continuity of the neural basis of personality, it is necessary to clarify the neural circuits that process personality in other animals and compare them with those in mammals. In birds, the DA system is anatomically and molecularly similar to that in mammals; however, the function of DRD4 remains largely unknown. In this study, we used chicks as model birds to reveal the expression regions of the DA neuron-related markers *tyrosine hydroxylase (TH), dopa decarboxylase (DDC), dopamine* β*-hydroxylase (DBH)*, and *DRD4*, as well as other *DRDs* throughout the forebrain. We found that *DRD4* was selectively expressed in the mitral cells of the olfactory bulb (OB). Furthermore, a detailed comparison of the expression regions of DA neurons and *DRD4* in the OB revealed a cellular composition similar to that of mammals. Our findings suggest that the animal personality gene *DRD4* is important for olfactory information processing in birds, providing a new basis for comparing candidate neural circuits for personality traits between birds and mammals.

## 1 Introduction

Personality, or animal personality, refers to consistent, genetically-based individual behavioral differences in humans and non-human animals that are stable across time and contexts (Gosling, 2001; van Oers and Mueller, 2010; Wolf and Weissing, 2012). The candidate gene that has been most intensively studied for its association with personality and considered the most promising is the *dopamine receptor D4* (*DRD4*) (Ebstein et al., 1996; Kluger et al., 2002; Munafò et al., 2008). Such associations between personality traits and *DRD4* polymorphisms are also known in animal personalities, including those of birds (Fidler et al., 2007), but the results of many studies have been ambiguous across species. To gain a deeper understanding of the phylogenetic continuity of the neural basis of personality, it is necessary to clarify the role of DRD4 in the brain function of each species.

In mammals, the dopamine (DA) system is involved in many physiological and higher cognitive functions and has attracted particular attention because of its involvement in human psychiatric disorders, including Parkinson's disease, schizophrenia, and addiction (Carlsson, 2006; Girault and Greengard, 2004; Iversen and Iversen, 2007; Klein et al., 2019). DA neurons are distributed in clusters in the central nervous system. These clusters are classified into two main groups: the A8-A10 cell groups located in the midbrain and the A11-A16 cell groups located in the forebrain (Dahlström and Fuxe, 1964; Bjorklund and Dunnett, 2007). Midbrain DA neuron cell groups project to a wide area of the forebrain, including the nucleus accumbens (NAc), amygdala, prefrontal cortex, and striatum. Recent research has begun to elucidate how DA influences neural circuits that regulate reward, motivation, and aversion at the cellular level (Hillarp et al., 1966; Bjorklund and Dunnett, 2007; Wise, 2004; Verharen et al., 2020; Yagishita, 2020, 2023). In contrast, forebrain DA neurons have local and spinal projections and are involved in the regulation of many physiological functions, including endocrine regulation (Ben-Jonathan and Hnasko, 2001). The released DA acts on its downstream targets, which is mediated by two families of DA receptors: the D1 and D2 receptor families. The D1 family includes DRD1 and DRD5 (also called DRD1B), and the D2 family includes DRD2, DRD3, and DRD4 (Yamamoto et al., 2015). The *DRD* genes encoding these DA receptors have different expression distributions in the brain, and each is thought to be involved in the regulation of various brain functions (Callier et al., 2003; Bentivoglio and Morelli, 2005). Studies using knockout mice for each DRD subtype combined with various behavioral assays are actively investigating the specific brain functions regulated by each DRD (Holmes et al., 2004). As for *DRD4* knockout mice, findings include increased motor hypersensitivity to ethanol, cocaine, methamphetamine, and methylphenidate (Rubinstein et al., 1997; Keck et al., 2013); reduced novelty seeking (Dulawa et al., 1999); enhanced anxiety-related behavior (Falzone et al., 2002); and effects on memory and learning (Ananth et al., 2019). However, no abnormalities in impulsivity were observed (Helms et al., 2008), and the effects on novelty seeking and anxiety-related behavior varied by sex (Thanos et al., 2015). Notably, behavioral abnormalities in response inhibition were observed in heterozygotes rather than in knockout mice (Young et al., 2011). Thus, although it has not yet been fully resolved, DRD4 appears to be related to novelty seeking, emotionality, and behavioral inhibition in mice.

In birds, the anatomical features of the DA system are similar to those in mammals (Reiner et al., 1994; Smeets and Reiner, 1994; Smeets and González, 2000; Reiner et al., 2004). Furthermore, hodological analyses have revealed that the input-output connections of the midbrain DA nuclei in birds are similar in extent to those in mammals (Csillag, 1999; Durstewitz et al., 1999; Mezey and Csillag, 2002; Balint and Csillag, 2007; Balint et al., 2011). The molecular characteristics of the avian DA system have also been thought to be largely conserved in mammals (Yamamoto and Vernier, 2011). However, recent findings suggest that they are not always highly conserved through vertebrates. For instance, birds have lost the *dopamine transporter* (*DAT*) gene from the genome, and the DAT function is compensated for by noradrenaline transporter (NAT) (Lovell et al., 2015; Fujita et al., 2022c). Therefore, the degree of conservation between the avian and mammalian DA systems requires careful re-examination. Although several studies have revealed the expression distribution of avian *DRD*s in the forebrain (Kubikova et al., 2010; Yamamoto et al., 2013), the region where *DRD4* is expressed remains unclear.

The most comprehensive study to date on the expression patterns of *DRD*s in the avian forebrain was conducted in songbirds. Kubikova et al. (2010) used probes for full-length songbird *DRD1, DRD2, DRD3, DRD4, DRD5*, and *DRD1C* (referred to as D1D in their study) to reveal the expression distribution across various songbird brain regions. Additionally, they performed cross-hybridization experiments on adult chicken brains using the same songbird probe sets (Kubikova et al., 2010). To date, no clear expression region for *DRD4* has been identified in avian brain regions except for the cerebellum. *DRD1E*, a member of the D1 family lost in mammals, has been identified in the chicken genome (Yamamoto et al., 2015). Therefore, to reveal the expression distribution of *DRD*s in the chick forebrain, we developed chicken-specific probes for all *DRD*s and conducted *in situ* hybridization (ISH) analysis. Furthermore, the distribution of avian DA neurons in the brain has been elucidated through histochemical and immunohistochemical techniques (Ikeda and Goto, 1971; Dube and Parent, 1981; Guglielmone and Panzica, 1984; Moons et al., 1994; Reiner et al., 1994, 2004). The distribution of cell bodies in the chick midbrain was previously revealed using ISH analysis of DA neuron-“related” markers. DA neurons are typically defined as *tyrosine hydroxylase* (*TH*)+/*dopa decarboxylase* (*DDC*)+/*dopamine* β*-hydroxylase* (*DBH)–* cell population (Fujita et al., 2022c), but those in the chick forebrain using DA neuron-“related” markers have not yet been clarified.

In this study, to better understand the function of DRDs in avians, we investigated the molecular anatomy of the chick forebrain DA system using ISH. We selected all DA receptor genes—*DRD1, DRD2, DRD3, DRD4, DRD5, DRD1C* (previously called *DRD1D*), and *DRD1E.—*along with the chick orthologs of DA neuron-related marker genes, *TH* and *dopa decarboxylase* (*DDC*), and as well as the noradrenergic neuron-related marker gene [*dopamine* β*-hydroxylase* (*DBH*)].

## 2 Materials and methods

### 2.1 Animals

Fertilized eggs of domestic chicks (*Gallus gallus domesticus*, Cobb strain) were purchased from a domestic company (3-M, Aichi Prefecture, Japan) and incubated at Teikyo University (Kaga, Itabashi-ku, Tokyo). Animal experiments were performed as described by Yamaguchi et al. (2008a,b). All chicks used in this study were 1-day-old chicks [post-hatched on day one (P1)]. All procedures were reviewed and approved by the Committee on Animal Experiments of Teikyo University and performed in accordance with the guidelines of the national regulations for animal welfare in Japan.

### 2.2 Histology

P1 chicks were anesthetized by intraperitoneal injection of a 1:1 solution of ketamine (10 mg/mL, ketalar-10, Sankyo Co., Tokyo, Japan) and xylazine (2 mg/mL, Sigma, St. Louis, MO, USA) at a dose of 0.40 mL per animal, and then transcardially perfused with 4% paraformaldehyde in 0.1 M phosphate-buffered saline (pH 7.5) (PFA-PBS). After perfusion, whole brains were surgically removed quickly and immediately placed in PFA-PBS, then immersed at 4°C for 1 day. The immersion solution was then changed to an 18% sucrose/PFA-PBS solution for cryoprotection and kept at 4°C for 2 days. The sucrose-substituted brains were then embedded in Tissue-Tek Optimal Cutting Temperature (OCT) compound (Sakura Finetechnical, Tokyo, Japan), rapidly frozen on dry ice, and stored at −80°C until further use. In this study, we used a total of 23 chicks, with the following distribution for each probe: 15 chicks for *TH*, 12 for *DDC*, 12 for *DBH*, 9 for *DRD1*, 11 for *DRD2*, 11 for *DRD3*, 12 for *DRD4*, 9 for *DRD5*, 9 for *DRD1C*, 9 for *DRD1E*, and 5 for *glutamate decarboxylase 2* (*GAD2*) (Supplementary Table S1). Frozen blocks, including brain samples, were cut into 18 μm-thick sections using a cryostat (Leica CM3050S or Leica CM1850, Leica Biosystems, Nußloch, Germany) and mounted on glass slides. The levels of serial coronal sections (A14.6-A4.6) were consistent with those of the chick brain atlas by Kuenzel and Masson (1988). If necessary, the chick brain atlas by Puelles et al. (2018) was used as a reference.

### 2.3 Selection of the chick orthologs of mammalian DA system genes

Chick orthologs of specific genes involved in the DA system, which are also present in mammals, were selected. Genes for DA production-related genes (*TH, DDC*, and *DBH*) and dopamine receptors (*DRD1, DRD2, DRD3, DRD4, DRD5, DRD1C*, and *DRD1E*) were included. The DNA and protein sequence similarities between chick and human genes (or zebrafish) for these DA system genes are presented in Supplementary Table S2. DA neurons are typically defined as neurons that express the rate-limiting enzymes *TH* and *DDC*, required for the stepwise synthesis of DA, but do not express *DBH*, the enzyme responsible for synthesizing noradrenaline from DA. There are two genes encoding *TH, TH1*, and *TH2* in vertebrates other than mammals. Here, *TH1* is referred to as *TH* in this study. Using BLAST search (https://blast.ncbi.nlm.nih.gov/Blast.cgi), we searched for sequence similarities between these DA production-related markers and those of other animals described in a previous study (Fujita et al., 2022c). A summary of these orthologous gene probes is presented in Table 1. When multiple transcript variants of an ortholog were registered in the database, probes were designed to detect all the variants. ISH was performed to analyze the expression patterns of the orthologs in the chick brain.

**Table 1:** Overview of DA system-related gene probes used in this study.

**Accession number**	**Gene symbol**	**Molecular characteristics**	**Probe position**	**Probe size (base)**	**Probe preparation**
NM_001144848.3	*DRD1*	GPCR	+23 ~+746	724	This study
NM_001113290.2	*DRD2*	GPCR	+7 ~+628	622	This study
XM_040646306.2	*DRD3*	GPCR	+5 ~+799	795	This study
NM_001142849.3	*DRD4*	GPCR	+107 ~+777	671	This study
XM_040670952.2	*DRD5*	GPCR	+201 ~+925	725	This study
NM_001142671.2	*DRD1C*	GPCR	+146 ~+806	661	This study
MK138990.1	*DRD1E*	GPCR	+417 ~+1,254	838	This study
XM_015282054.4	*GAD2*	enzyme	+97 ~+788	692	This study
NM_204805.2	*TH*	enzyme	+119 ~+835	717	Fujita et al. (2022c)
XM_419032.7	*DDC*	enzyme	+38 ~+758	721	Fujita et al. (2022c)
XM_040686111.1	*DBH*	enzyme	+676 ~+1,429	754	Fujita et al. (2022c)

*DBH*, dopamine beta-hydroxylase*; DDC*, dopa decarboxylase*; DR, dopamine receptor; GAD2, glutamate decarboxylase 2; GPCR*, G protein-coupled receptor; *TH*, tyrosine hydroxylase.

### 2.4 cDNA cloning and RNA probe preparations

Total RNA was extracted from a chick brain using TRIzol Reagent (Invitrogen, Carlsbad, CA, USA) and subjected to a reverse-transcription (RT) reaction using an oligo (dT) primer with SuperScript III kit (Invitrogen, Carlsbad, CA, USA) according to the manufacturer's protocol. RT polymerase chain reaction (RT-PCR) was performed using the following gene-specific primer (forward and reverse) pairs: *DRD1:*5′-TGGATGGAGAAGGGTTGCTT-3′ and 5′- CTGCTTCTGTTGCCACTTGT-3,' respectively; *DRD2:*5′-CCCCTGAATCTATCCTGGTACA-3′ and 5′-AGATCTGCACGTACACCAGC-3,' respectively; *DRD3:*5′-ATGTCATGATGTGCACAGCC-3′ and 5′-GGGCGTTGAGGATGTGAATC-3,' respectively; *DRD4:*5′-TCGTCCTCATCCTGCTCATC-3′ and 5′-GTGGGCATAAGGGTGGTACT-3,' respectively; *DRD5:*5′-CATCTTCATCGTGTCGCTGG-3′ and 5′-TGATGGAGGACTTGAGGCTG-3,' respectively; *DRD1C:* 5′-ACTGGTTTGTGCTGTCGTTG-3′ and 5′-TGACAGAAAGGTAGCAGGCA-3,' respectively; *DRD1E:* 5′-CAACCCCTTCTGCTACGAGA-3′ and 5′-GGCTGCTTTGTACTCCACTG-3,' respectively; *GAD2:* 5′-GCACAGAAGTTCACCGGAG-3′ and 5′-GGGAACATCTTGAAACGTGC-3,' respectively. The resulting PCR amplicons were subcloned into a pGEM-T Easy Vector (Promega, Madison, WI, USA). Subcloning of the target sequences was confirmed using Sanger sequencing. For the *TH, DDC*, and *DBH* probes, we used plasmids prepared in a previous study (Fujita et al., 2022c). All plasmids containing cDNA fragments were amplified by PCR with M13 primer pairs, and amplicons containing the T7 and SP6 promoter sites were purified using a PCR purification kit (Qiagen, Valencia, CA, USA). The digoxigenin (DIG)-labeled sense and antisense RNA probes were prepared by *in vitro* transcription using a DIG RNA labeling kit (Roche, Basel, Switzerland).

### 2.5 ISH

ISH experiments were performed according to the method described by Fujita et al. (2019) with minor modifications. Briefly, brain sections on glass slides were fixed in 4% PFA-PBS. After incubation in 10 μg/ml proteinase K in 10 mM Tris-HCl and 1 mM EDTA, the specimens were post-fixed for 10 min in PFA–PBS, treated with 0.2 M HCl for 10 min, washed in PBS, and treated with 0.25% acetic anhydride in 0.1 M triethanolamine (pH 7.5) for 10 min. Hybridization was performed with DIG-labeled RNA probes at 70°C for 19 h 30 min to 21 h 10 min. The size of the probes used is shown in Table 1. After stringent washing, the hybridized probes were immunohistochemically detected using an alkaline phosphatase-conjugated anti-DIG antibody (1:1,000, Roche, Basel, Switzerland). To visualize the signals, a chromogenic reaction with a nitro blue tetrazolium/5-bromo-4-chloro-3-indolyl phosphate was performed at room temperature (~24°C) for 16 h 15 min to 20 h 10 min for all probes. Sense probes were used as negative controls in all the experiments.

### 2.6 Imaging and data processing

Sections on each slide were semi-automatically acquired as digital slides using a NanoZoomer 2.0HT or NanoZoomer XR system (Hamamatsu Photonics, Shizuoka, Japan). Digital photographs of the regions of interest were manually extracted from digital slides using the NDP.view2 software (Hamamatsu Photonics, Shizuoka, Japan). The entire extracted images were converted to 8-bit images, and the brightness and contrast of the entire extracted images were adjusted using ImageJ (https://imagej.nih.gov/ij/).

## 3 Results

### 3.1 Expression of *TH, DDC*, and *DBH* in the chick forebrain

**Figure 1:**
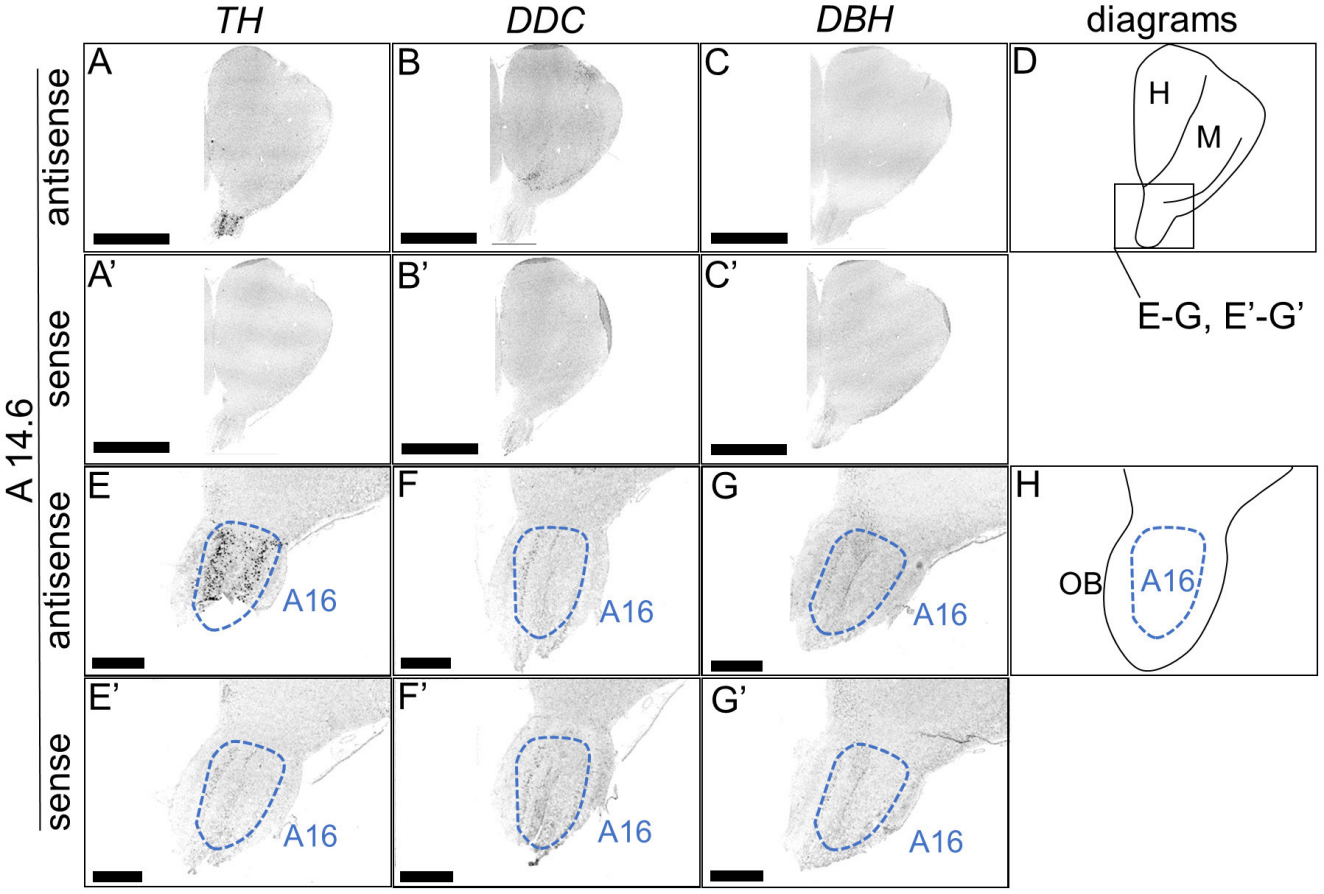
*In situ* hybridization of *TH, DDC*, and *DBH* in the P1 chick rostral forebrain. Digoxigenin-labeled RNA probes, both antisense [*TH*, **(A, E)**, *DDC*, **(B, F)**, and *DBH*, **(C, G)**] and sense [*TH*, **(A', E')**, *DDC*, **(B', F')**, and *DBH*, **(C', G')**], were used for *in situ* hybridization in coronal sections of P1 chick forebrains, corresponding level A14.6 in the Kuenzel and Masson's chick atlas (Kuenzel and Masson, 1988). Signal reproducibility was verified across multiple chicks at the same level, with representative images from neighboring sections provided. **(E**–**G**, **E'**–**G')** present magnified views of the forebrain region indicated by the box in **(D)**. Diagrams of coronal sections depicted in **(A, E)** are shown in **(D, H)**, respectively. A16, A16 cell group; H, hyperpallium; M, mesopallium; OB, olfactory bulb. Scale bars = 2.5 mm **(A–C, A'–C')** and 500 μm **(E–G, E'–G')**.

**Figure 2:**
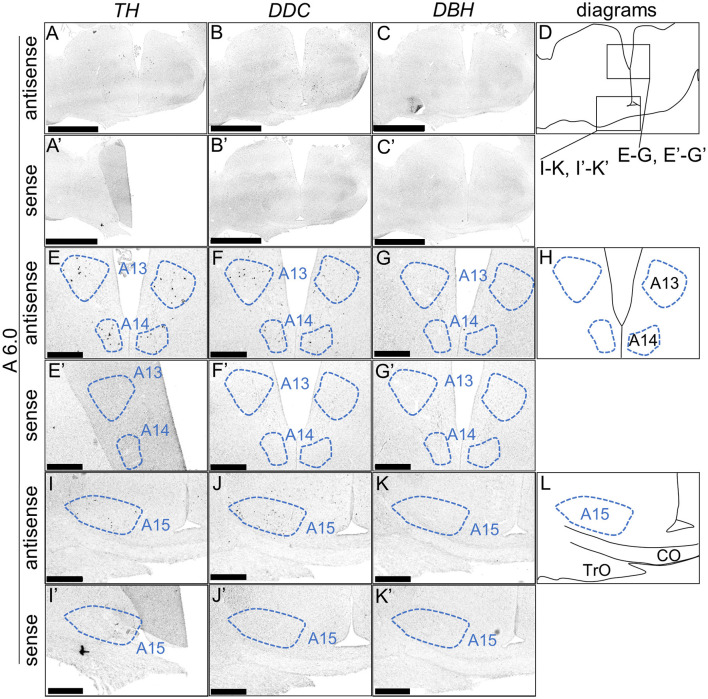
*In situ* hybridization of *TH, DDC*, and *DBH* in the P1 chick diencephalon. Digoxigenin-labeled RNA probes, both antisense [*TH*, **(A, E, I)**, *DDC*, **(B, F, J)**, and *DBH*, **(C, G, K)**] and sense [*TH*, **(A', E', I')**, *DDC*, **(B', F', J')**, and *DBH*, **(C', G', K')**], were used for *in situ* hybridization in coronal sections of P1 chick forebrains, corresponding to level A6.0 in the Kuenzel and Masson's chick atlas (Kuenzel and Masson, 1988). Signal reproducibility was verified across multiple chicks at the same level, with representative images from neighboring sections provided. **(E**–**G, I–K, E'**–**G', I'–K')** present magnified views of the diencephalon region indicated by the box in **(D)**. Diagrams of coronal sections depicted in **(A, E, I)** are shown in **(D**, **H, L)**, respectively. A13, A13 cell group; A14, A14 cell group; A15, A15 cell group; CO, chiasma opticum; TrO, tractus opticus. Scale bars = 2.5 mm **(A–C, A'–C')** and 500 μm **(E–G, I–K, E'–G', I'-K')**.

**Figure 3:**
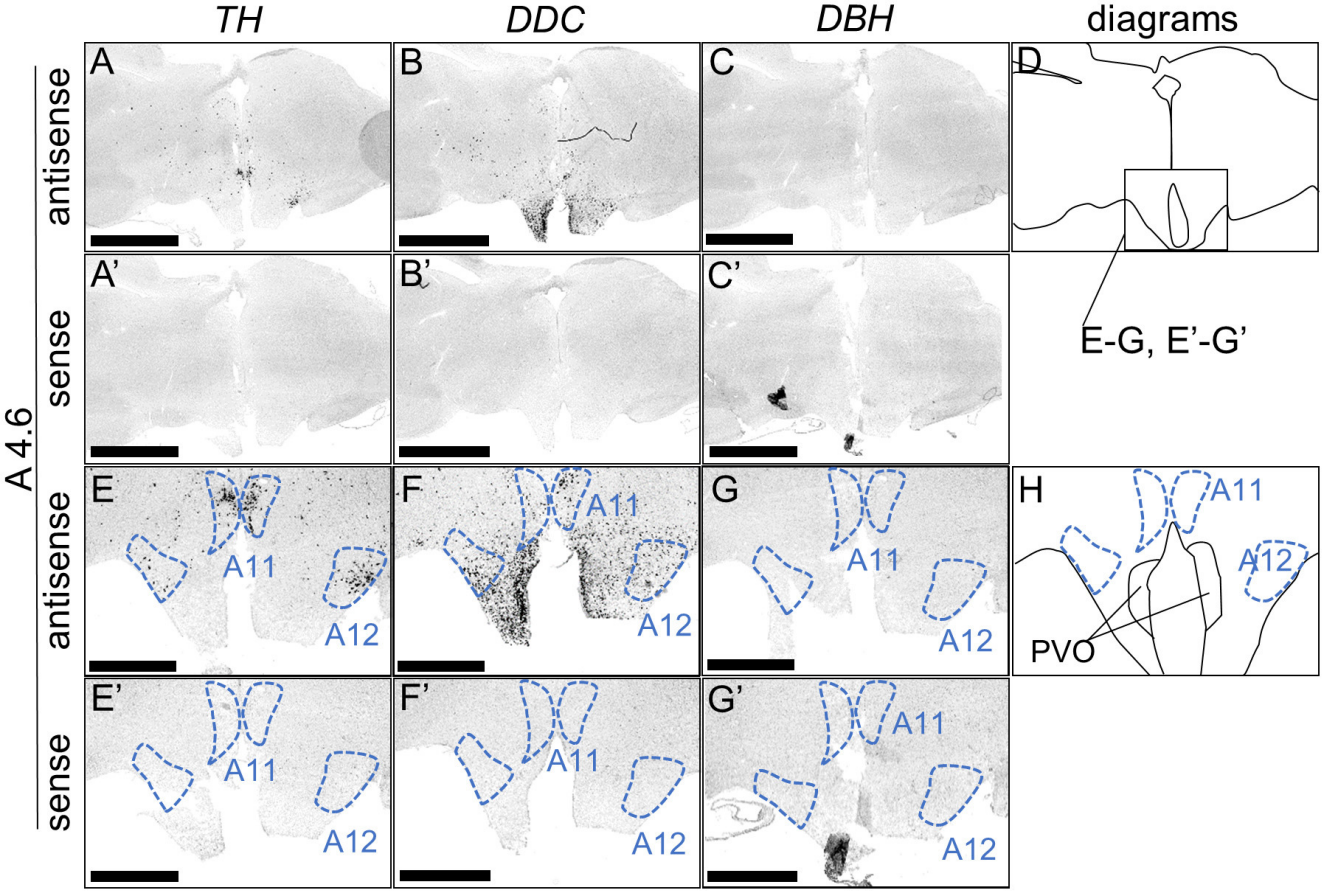
*In situ* hybridization of *TH, DDC*, and *DBH* in the P1 chick hypothalamus. Digoxigenin-labeled RNA probes, both antisense [*TH*, **(A, E)**, *DDC*, **(B, F)**, and *DBH*, **(C, G)**] and sense [*TH*, **(A', E')**, *DDC*, **(B', F')**, and *DBH*, **(C', G')**], were used for *in situ* hybridization in coronal sections of P1 chick hypothalamus, corresponding to the A4.6 level in the Kuenzel and Masson's chick atlas (Kuenzel and Masson, 1988). Signal reproducibility of the signals was confirmed across multiple chicks at the same level, with representative images from neighboring sections provided. **(E**–**G**, **E'**–**G')** present magnified views of the hypothalamus region indicated by the box in **(D)**. Diagrams of coronal sections depicted in **(A**, **E)** are shown in **(D**, **H)**, respectively. A11, A11 cell group; A12, A12 cell group; PVO, paraventricular organ. Scale bars = 2.5 mm **(A–C, A'–C')** and 1 mm **(E–G, E'–G')**.

We performed ISH analysis to investigate the expression patterns of the DA neuron-related markers—*TH, DDC*, and *DBH*—using adjacent sections from A14.6 to A4.6 in the forebrain of chicks on post-hatched day 1(P1). We detected cells showing signals of *TH* in the A16 (Figures 1A, A'), A15 (Figures 2A, I, A', I'), A14 and A13 (Figures 2A, E, A', E'), A12 and A11 (Figures 3A, E, A', E') regions. *DDC*-positive cells were detected in the A15 (Figures 2B, J, B', J'), A14 and A13 (Figures 2B, F, B', F'), PVO, A12 and A11 (Figures 3B, F, B', F') regions. We found that the expression patterns of *TH* and *DDC* in A15-A11 in the chick were similar, confirming the absence of *DBH* signals.

**Figure 4:**
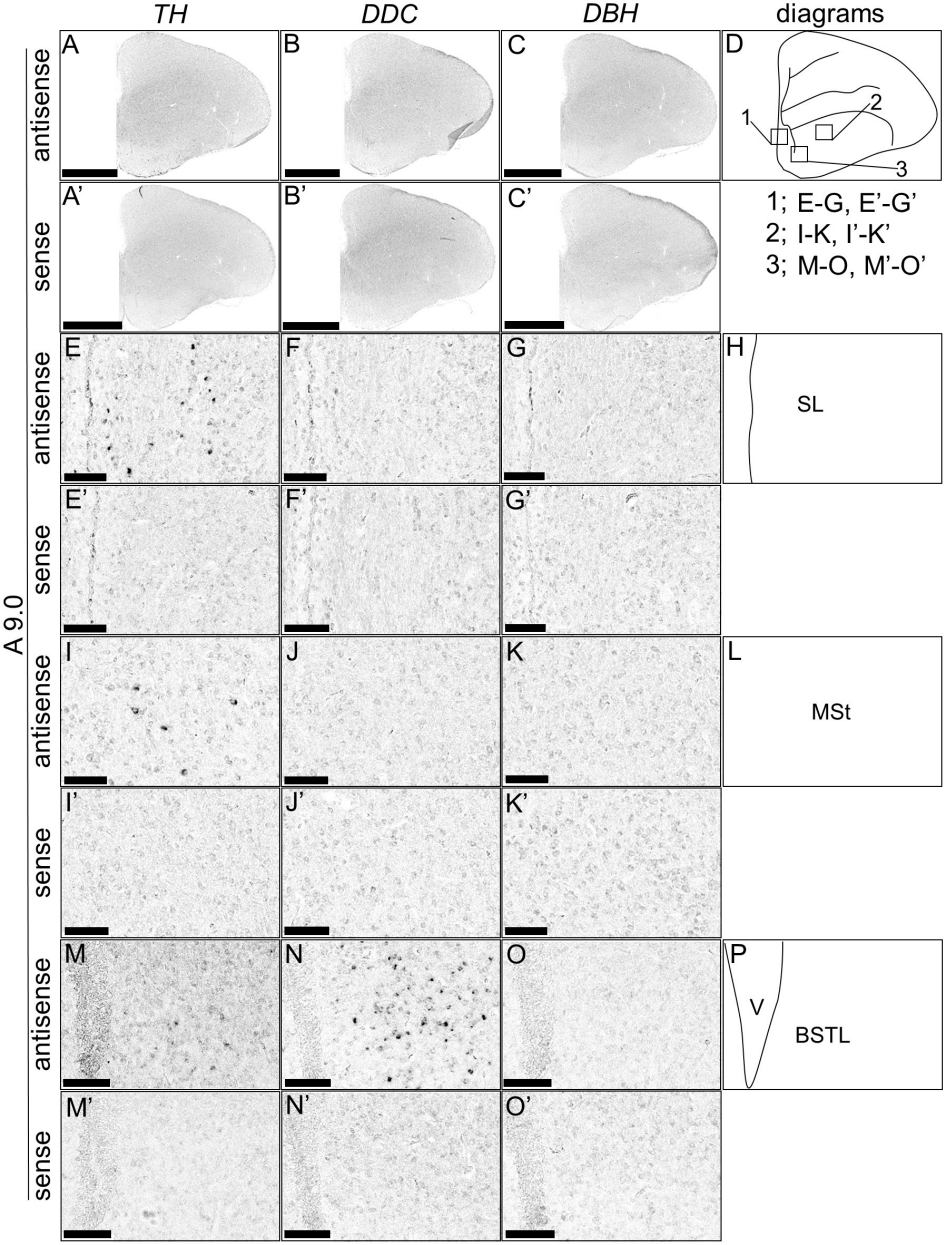
*In situ* hybridization of *TH, DDC*, and *DBH* in the P1 chick A9.0 level forebrain. Digoxigenin-labeled RNA probes, both antisense [*TH*, **(A, E, I, M)**, *DDC*, **(B, F, J, N)**, and *DBH*, **(C, G, K, O)**] and sense [*TH*, **(A', E', I', M')**, *DDC*, **(B', F', J', N')**, and *DBH*, **(C', G', K', O')**], were used for *in situ* hybridization in coronal sections of P1 chick forebrains, corresponding to level A9.0 in the Kuenzel and Masson's chick atlas (Kuenzel and Masson, 1988). Signal reproducibility was verified across multiple chicks at the same level, with representative images from neighboring sections provided. **(E–G, I–K, M–O, E'–G', I'–K', M'–O')** present magnified views of the forebrains region indicated by the box in **(D)**. Diagrams of coronal sections depicted in **(A**, **E**, **I**, **M)** are shown in **(D, H, L, P)**, respectively. BSTL, bed nucleus of the stria terminalis; MSt, medial striatum; SL, lateral septal nucleus. Scale bars = 2.5 mm (**A–C**, **A'–C'**) and 100 μm **(E–G, I–K, M–O**, **E'–G', I'-K', M'-O')**.

**Figure 5:**
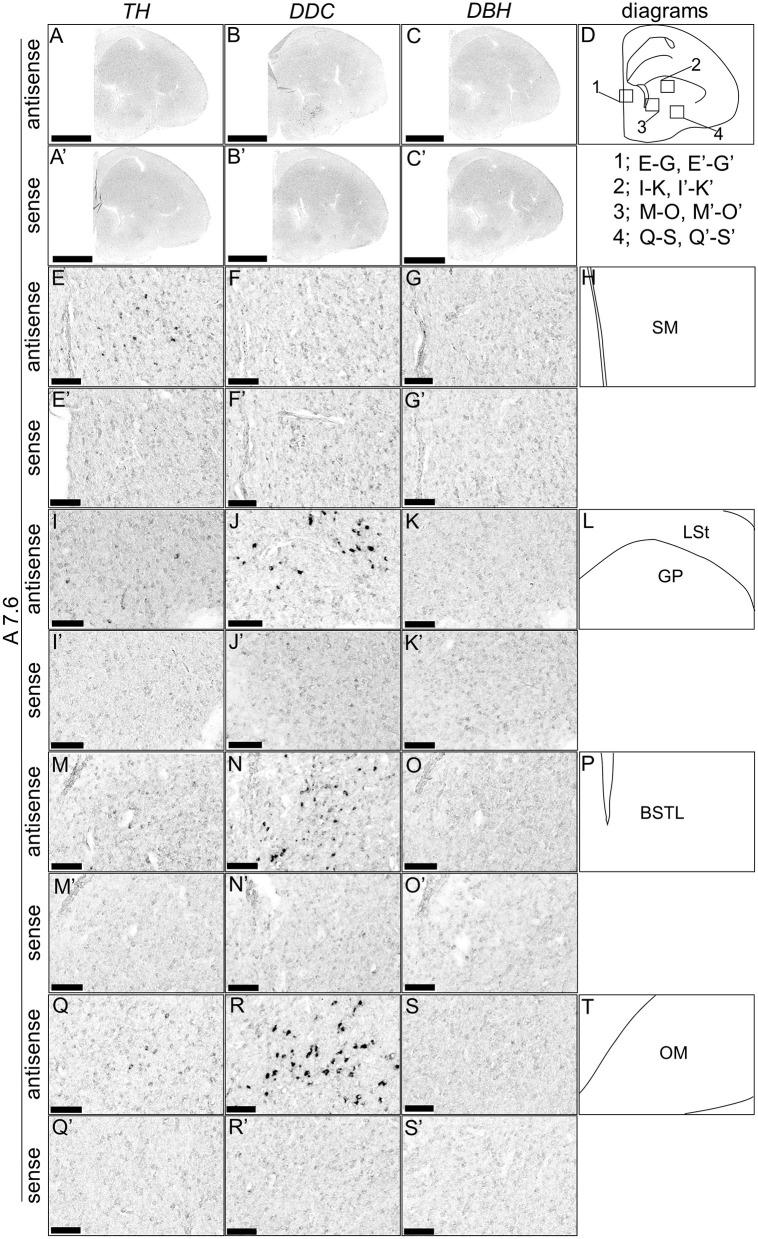
*In situ* hybridization of *TH, DDC*, and *DBH* in the P1 chick A7.6 level forebrain. Digoxigenin-labeled RNA probes, including antisense [*TH*, **(A, E, I, M, Q**), *DDC*, (**B, F, J, N, R**), and *DBH*, (**C, G, K, O, S)**] and sense [*TH*, **(A', E', I', M', Q')**, *DDC*, **(B', F', J', N', R')**, and *DBH*, **(C', G', K', O', S')**] probes, were used for *in situ* hybridization in coronal sections of P1 chick forebrains, corresponding to the A7.6 level in the Kuenzel and Masson's chick atlas (Kuenzel and Masson, 1988). Signal reproducibility was verified across multiple chicks at the same level, with representative images from adjacent sections provided. **(E**–**G, I–K, M–O, Q–S**, **E'**–**G', I'–K', M'–O', Q'–S')** present magnified views of the forebrains region indicated by the box in **(D)**. Diagrams of coronal sections presented in **(A, E, I, M, Q)** are shown in **(D, H, L, P, T)**, respectively. BSTL, bed nucleus of the stria terminalis; GP, globus pallidus; LSt, lateral striatum; OM, occipito-mesencephalic tract; SM, medial septal nucleus. Scale bars = 2.5 mm **(A–C**, **A'–C')** and 100 μm **(E–G, I–K, M–O, Q–S**, **E'–G', I'–K', M'–O', Q'–S')**.

**Figure 6:**
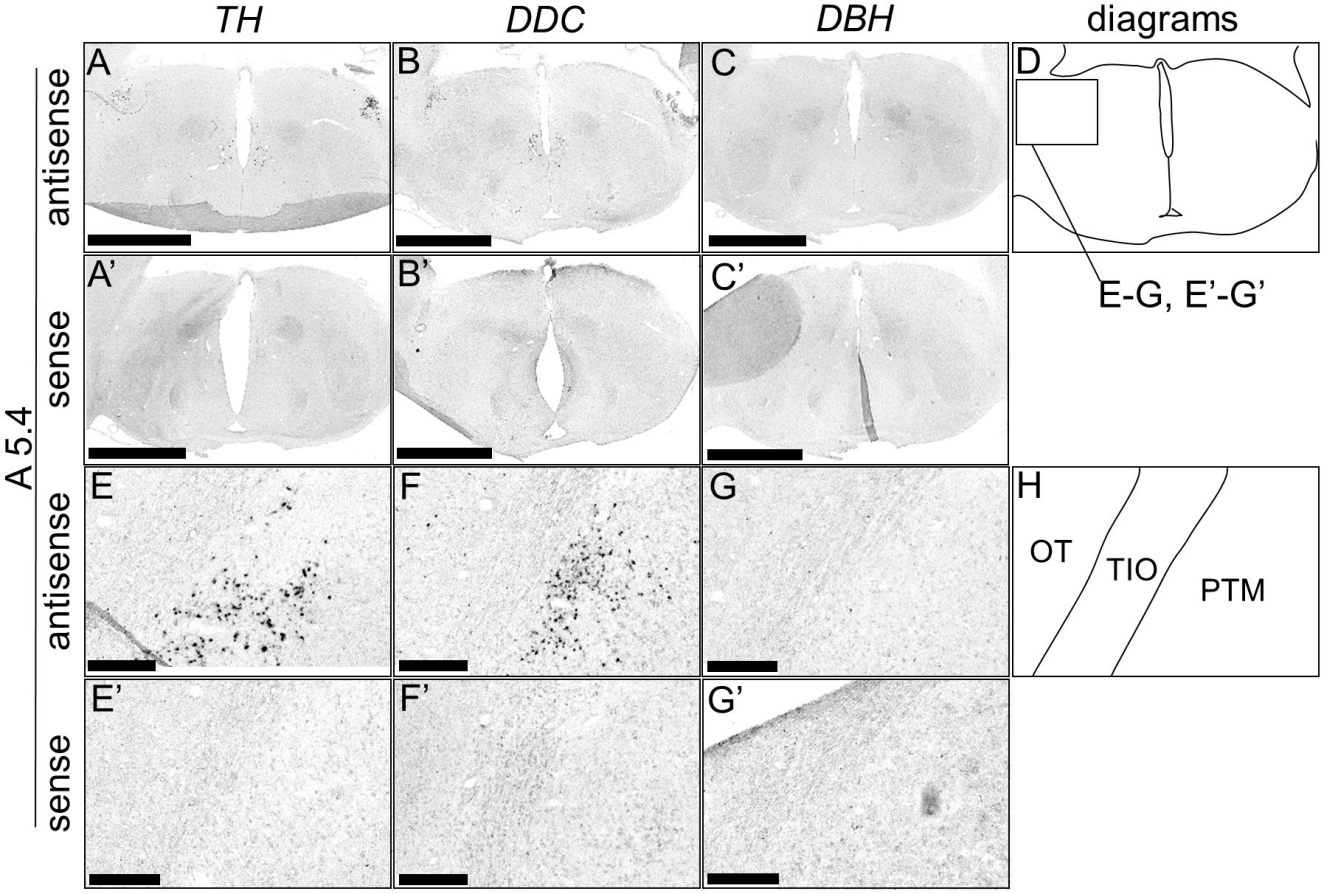
*In situ* hybridization of *TH, DDC*, and *DBH* in the P1 chick A5.4 level forebrain. Digoxigenin-labeled RNA probes, both antisense [*TH*, **(A, E)**, *DDC*, **(B, F)**, and *DBH*, **(C, G)**] and sense [*TH*, **(A', E')**, *DDC*, **(B', F')**, and *DBH*, **(C', G')**], were used for *in situ* hybridization in coronal sections of P1 chick forebrains, corresponding to level A5.4 of the Kuenzel and Masson's chick atlas (Kuenzel and Masson, 1988) was confirmed in multiple chicks at the same level, and representative images of the neighboring sections are shown. **(E**–**G**, **E'–G')** Magnified views of forebrains in the box in **(D)**. **(D, H)** Diagrams of coronal sections are shown in **(A, E)**. OT, optic tectum; PTM, nucleus pretectalis medialis; TIO, tractus ishmo-opticus. Scale bars = 2.5 mm **(A–C**, **A'–C')** and 250 μm **(E–G**, **E'–G')**.

Although typical DA neurons are defined by the expression of *TH* and *DDC* but not *DBH*, neurons exhibiting only some of the characteristics of DA neurons have been identified in mammals (Bjorklund and Dunnett, 2007; Ugrumov, 2009). Specifically, neurons expressing only *TH* or *DDC* monoenzymatically, but not *DBH*, are considered to partially express the DA neuron phenotype. In this study, we detected such neurons in the forebrain of chicks. *TH*-positive cells were detected in the substantia nigra (SN), medial striatum (MSt), bed nucleus of the stria terminalis, lateral part (BSTL), septum mediale (SM), globus pallidus (GP), olfactory bulb (OB), and periventricular thalamic nuclei (PTM) as shown in Figures 4A, E, I, M, A', E', I', M', 5A, E, I, M, Q, A', E', I', M', Q', and 6A, E, A', E', respectively. Cells with signals of *DDC* were detected in the BSTL, lateral striatum (LSt), GP, occipito-mesencephalic tract (OM), and PTM (Figures 4B, N, B', N', 5B, J, N, R, B', J', N', R', and 6B, F, B', F'), respectively. No *DBH* signal was detected in these regions. Additionally, *TH* and *DDC* signals were observed in nearby areas, though their expression patterns differed.

### 3.2 Expression of *DRD1, DRD2, DRD3, DRD4, DRD5, DRD1C*, and *DRD1E* in the chick forebrain

**Figure 7:**
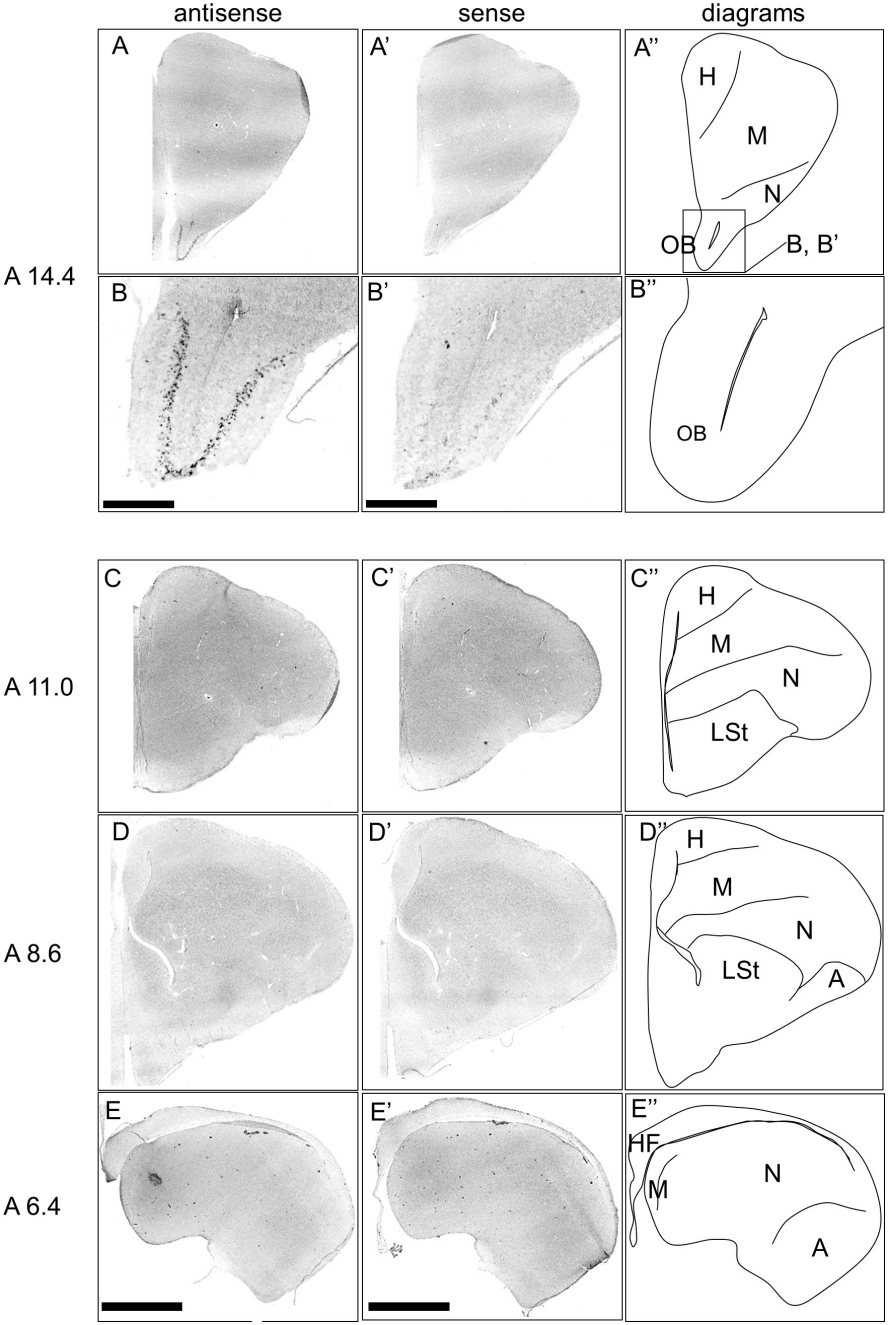
*In situ* hybridization of *DRD4* in P1 chick telencephalons. Digoxigenin-labeled RNA antisense RNA probes, both antisense **(A–D)** and sense **(A'–D')** *DRD4* probes were used for *in situ* hybridization in coronal sections of the P1 chick telencephalon. Signal reproducibility was verified across multiple chicks at the same level, with representative images provided. Diagrams of coronal sections are shown in the rightmost **(A–D”)**. Levels of sections (A 14.4 to A 6.4) were in accordance with those mentioned in Kuenzel and Masson's chick atlas (Kuenzel and Masson, 1988). **(B, B')** present magnified views of the olfactory bulbs region indicated by the box in (**A”**). A, arcopallium; H, hyperpallium; HF, hippocampal formation; LSt, lateral striatum; M, mesopallium; N, nidopallium; OB, olfactory bulb. Scale bars = 2.5 mm **(A, C–E, A', C'–E')** and 500 μm **(B, B')**.

First, the expression pattern of *DRD4* was examined in sections A14.4 to A6.4 in the P1 chick forebrains (Figure 7). Signals were generally poor, consistent with findings from a previous study (Kubikova et al., 2010). However, clear and characteristic signals were detected in the OB (Figures 7A, A', B, B').

We examined the expression pattern of *DRD1* in sections A14.0 to A6.2 in the P1 chick forebrains (Supplementary Figure S1). Signals were detected in the striatum (Supplementary Figures S1B, C, B', C'), lateral nidopallium, dorsal arcopallium (Supplementary Figures S1D, D'), and the hippocampal formation (HF), particularly in the dorsolateral region (Supplementary Figures S1D, D'). We examined the expression pattern of *DRD2* in sections A14.4 to A5.8 in the P1 chick forebrains (Supplementary Figure S2), and signals were detected in the striatum, except for GP (Supplementary Figures S2B, C, B', C'). We examined the expression pattern of *DRD5* in sections A14.0 to A6.4 in the P1 chick forebrains (Supplementary Figure S3). Signals were detected in the mesopallium (Supplementary Figures S3A, B, C, A', B', C'), striatum (Supplementary Figures S3B, C, B', C'), and HF, especially the V-shape region (Supplementary Figures S3D, D'). We examined the expression pattern of *DRD1C* in sections A14.0 to A5.8 in the P1 chick forebrains (Supplementary Figure S4). Signals were detected in the mesopallium (Supplementary Figures S4B, C, D, B', C', D'), hyperpallium (Supplementary Figures S4C, C'), and HF, especially the V-shape region (Supplementary Figures S4C, D, C', D'). The *DRD1, DRD2, DRD5*, and *DRD1C* expression sites were consistent with those of previous studies (Kubikova et al., 2010; Yamamoto et al., 2013), suggesting that the expression sites of these receptors are generally complete in P1 chicks.

**Figure 8:**
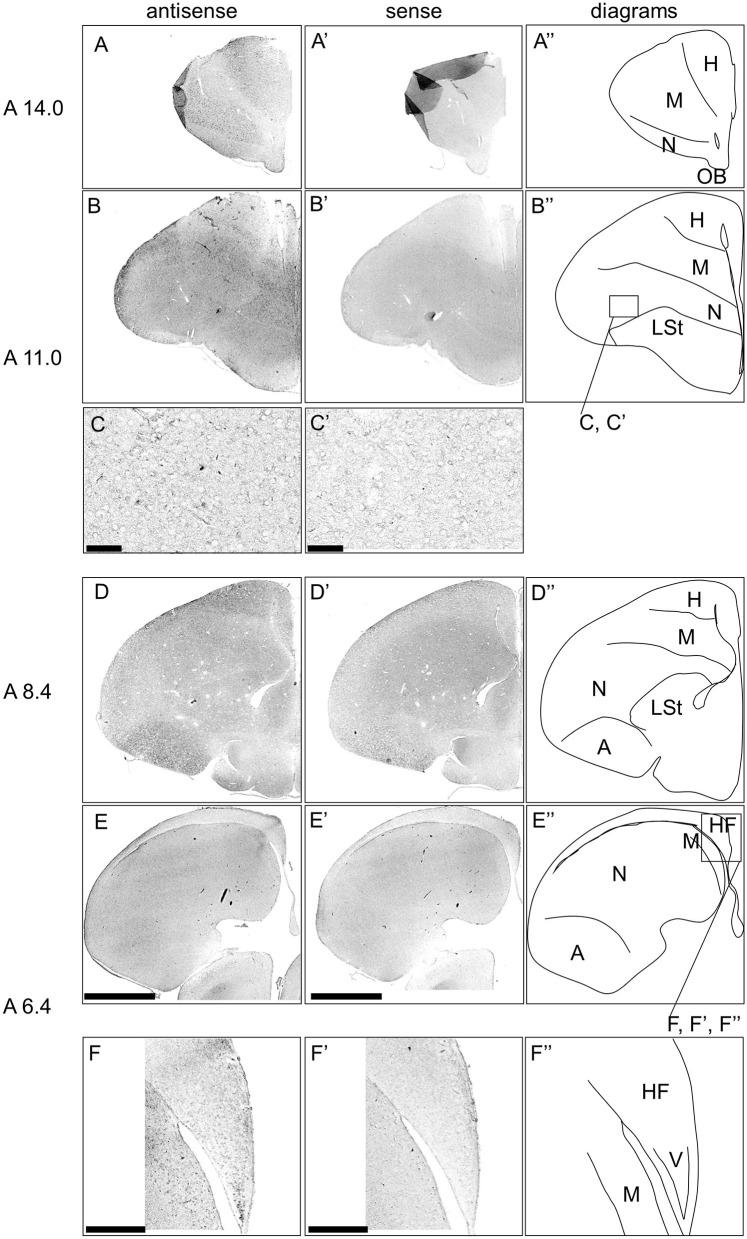
*In situ* hybridization of *DRD3* in P1 chick telencephalons. Digoxigenin-labeled RNA antisense **(A–F)** and sense **(A'–F')** *DRD3* probes were used for *in situ* hybridization in coronal sections of the P1 chick telencephalon. Reproducibility of signals was confirmed in multiple chicks at the same level, and representative images are shown. Diagrams of coronal sections are shown in the rightmost **(A”–F”)**. The levels of sections (A 14.0–A 6.4) were in accordance with those mentioned in Kuenzel and Masson's chick atlas (Kuenzel and Masson, 1988). **(C, C')** show magnified views of the entopallium **(C, C')** regions indicated by the box in **(B”)**, and **(F, F')** show magnified views of the HF **(F, F')** regions indicated by the box in **(E”)**. A, arcopallium; H, hyperpallium; HF, hippocampal formation; LSt, lateral striatum; M, mesopallium; N, nidopallium; OB, olfactory bulb; V, V-shape region. Scale bars = 2.5 mm **(A–D, A'–D')**, 100 μm **(E, E')**, and 500 μm **(F, F')**.

Next, we examined the expression pattern of *DRD3* in sections A14.0 to A6.4 in the P1 chick forebrains (Figure 8). Signals were detected in the dorsal and ventral mesopallium (Figures 8A, B, D, E, A', B', D', E'), hyperpallium, especially the interstitial nucleus of the hyperpallium apicale (IHA) (Figures 8A, B, A', B'), and the intermediate arcopallium (Figures 8D, D'), which is consistent with a previous study (Kubikova et al., 2010). Moreover, we detected very sparse signals in the entopallium (Figures 8B, C, B', C') and the HF, especially the V-shape region (Figures 8E, F, E', F').

Finally, we examined the expression pattern of *DRD1E* in sections A14.0 to A6.4 in the P1 chick forebrains and did not obtain clear signals (Supplementary Figure S5).

### 3.3 Comparison of the *TH, DRD4*, and *GAD2* in the chick OB

**Figure 9:**
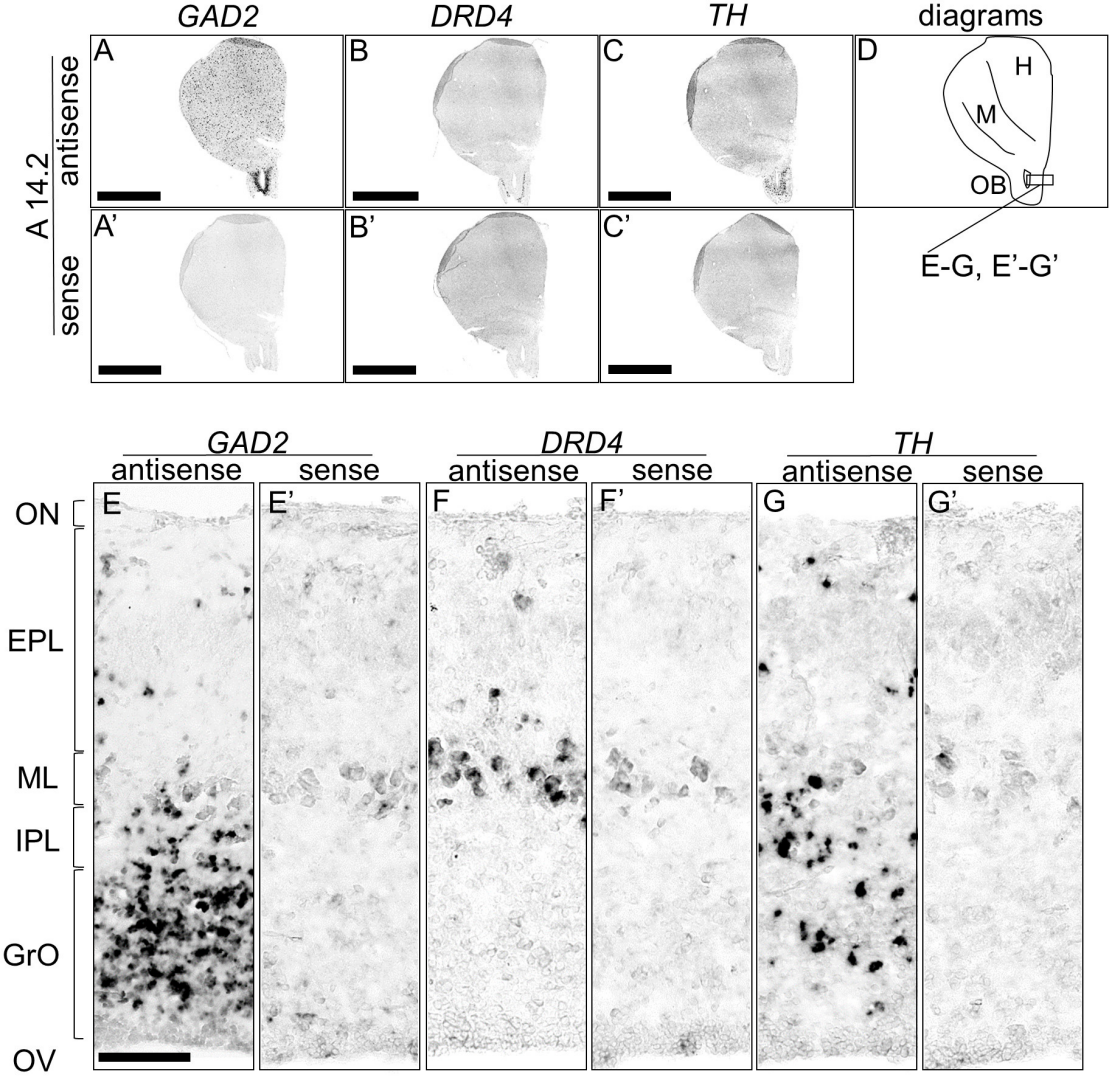
Comparison of *GAD2, DRD4*, and *TH* expression patterns in the olfactory bulb in the P1 chick rostral forebrain. Digoxigenin-labeled RNA probes, both antisense [*GAD2*, **(A, E)**, *DRD4*, **(B, F)**, and *TH*, **(C, G)**] and sense [*GAD2*, **(A', E')**, *DRD4*, **(B', F')**, and *TH*, **(C', G')**], were used for *in situ* hybridization in coronal sections of P1 chick rostral forebrains, corresponding to level A14.2 of the Kuenzel and Masson's chick atlas (Kuenzel and Masson, 1988). Signal reproducibility was confirmed across multiple chicks at the same level. Representative images of neighboring sections are shown. **(E–G, E'–G')** present magnified views of the olfactory bulb region indicated by the box in **(D)**. Diagram of coronal sections depicted in **(A)** is shown in **(D)**. EPL, external plexiform layer; GrO, granule cell layer of the olfactory bulb; H, hyperpallium; IPL, internal plexiform layer; M, mesopallium; ML, mitral cell layer; OB, olfactory bulb; ON, olfactory nerve layer; OV, olfactory ventricle. Scale bars = 2.5 mm **(A–C, A'–C')** and 100 μm **(E–G, E'–G')**.

We detected signals of *TH* and *DRD4* in the OB. In mammals, the DA neurons in the OB are known to be γ-aminobutyric acid (GABA) ergic interneurons (Cave and Baker, 2009). To further explore the DA system in the avian OB, we compared the expression patterns of *TH, DRD4*, and *GAD2*, a marker for GABAergic neurons that encodes the enzyme for GABA synthesis (Figure 9). *GAD2* signals were densely distributed in the glomerular layer (GrO), moderately in the inner plexiform layer (IPL), and sparsely in the external granular layer (EPL) (Figures 9A, E, A', E'). *DRD4* signals were specifically detected in the mitral cell layer (ML) (Figures 9B, F, B', F'), while *TH* signals were moderately distributed in the GrO and IPL and sparsely in the EPL (Figures 9C, G, C', G').

## 4 Discussion

There is a lot of evidence showing the association between *DRD4* polymorphism and animal personality and physical condition in birds (Fidler et al., 2007; Flisikowski et al., 2009; Korsten et al., 2010; Gillingham et al., 2012; Mueller et al., 2013; Garamszegi et al., 2014; Mueller et al., 2014; Timm et al., 2015; van Dongen et al., 2015; Holtmann et al., 2016). *DRD4* polymorphism was also detected in chickens (Sugiyama et al., 2004). However, the evidence is inconsistent depending on the bird population and the conditions of behavior measurement (Mueller et al., 2013; Edwards et al., 2015; Riyahi et al., 2015, 2017; Timm et al., 2019; Mai et al., 2023). Recently, Silva *et al*. demonstrated, through a behavioral pharmacological study on D1 and D2 receptors, that personality-related behaviors in birds can be influenced by the manipulation of DA signaling (Silva et al., 2020). However, since no drugs with high selectivity for DRD4 alone among D2 receptors are available, the neural functions of DRD4 in birds are unknown; therefore, the mechanism by which DRD4 affects animal personality in birds is unclear. In the present study, we clarified the distribution of DA neurons and the expression regions of *DRD*s in the chick forebrain to obtain clues about the function of the animal personality gene *DRD4* in the avian brain.

First, we revealed the expression patterns of the DA neuron–related markers *TH, DDC*, and *DBH* in the chick forebrain. Typically, DA neurons can be defined as a *TH*+/*DDC*+/*DBH*– cell population. We detected neuron populations with such characteristics in the chick A11 to A15 regions. This characteristic is similar to that observed in the avian forebrain using anti-DA or anti-TH antibodies (Bailhache and Balthazart, 1993; Moons et al., 1994; Reiner et al., 1994). In contrast, *TH*+/*DDC*–/*DBH*– neurons were detected in A16 region. Generally, neurons with these characteristics may be immature and have only partial properties of DA neurons or maybe a DOPAergic neuron population. Nevertheless, previous studies using anti-DA antibodies in other vertebrates have shown that DA is abundant in the OB, and because there is known species variation in *DDC* expression, these *TH*+/*DDC–/DBH–* neuron populations are proposed to be DA neurons (Reiner et al., 1994; Cave and Baker, 2009). *TH*–/*DDC*+/*DBH*– neurons were detected in the PVO (Figure 3). This population is absent in mammals. Previous studies have revealed DA immunopositivity and TH immunonegativity, suggesting that these neurons do not synthesize DA but rather accumulate it (Smeets and Reiner, 1994). In contrast, recent studies have shown that in vertebrates other than mammals, there are two genes encoding TH, *TH1* and *TH2*, which have different immunogenicities. However, *TH2* has been lost from the mammalian genome (Yamamoto et al., 2010). Furthermore, *TH2* is expressed in the corresponding PVO population in fish, amphibians, and birds (chicken), strongly suggesting that this population has the ability to synthesize and secrete DA (Yamamoto et al., 2011; Xavier et al., 2017). Our data (a *TH*–/*DDC*+/*DBH*– neuron population in the PVO) support the latter possibility (Figure 3). Future studies should focus on the biochemical characterization of TH2 enzyme activity in chicken PVO. Taken together, our data are consistent with the previous findings on avian DA systems.

In addition to the classical DA population, regions with *TH*+/*DDC–/DBH–* and *TH–*/*DDC*+/*DBH–* characteristics were detected in chick forebrain (Figures 4–6). Many such monoenzymatic DA neuron populations are well-documented in mammals, with distribution across various regions, including the striatum and BSTL (Ugrumov, 2009; Bupesh et al., 2014). Our results demonstrate that monoenzymatic DA neuron populations are distributed in multiple brain regions, including the striatum and BSTL, in the chick forebrain. This suggests that the presence of monoenzymatic DA neurons in these regions may be conserved between mammals and birds. Previous studies have suggested that these populations contain immature DA neurons and that in the rat arcuate nucleus, TH+/DDC– and TH–/DDC+ monoenzymatic neuron populations are present in close proximity to each other, suggesting that they synthesize and secrete DA in a coordinated manner (Ugrumov, 2009). In our data, we observed such a tendency, for example, in the *TH*+/*DDC–/DBH–* and *TH–*/*DDC*+/*DBH–* populations in the chick PTM (Figure 6). The existence of such populations in the forebrain may not only be evolutionarily conserved between birds and mammals but also in birds, where such a cooperative relationship exists.

Next, we investigated the expression patterns of all *DRD*s, including *DRD1, DRD2, DRD3, DRD4, DRD5*, and *DRD1C*, in the chick forebrain. Our findings clearly demonstrate, for the first time, that *DRD4* is specifically expressed in the mitral cells of the olfactory bulb, a region where its expression was previously unknown (Figures 7, 9). In mammals, mitral cells are output neurons that transmit information from the olfactory nerve to outside the OB and are modulated by DA (Cave and Baker, 2009). DA modulation of mitral cells *via* DRD4 may also play an important role in olfactory information processing in birds. The relationship between olfactory information processing and animal personality is currently unknown, and neither how mitral cells process external information may reflect animal personality. Birds have sophisticated sensory systems, and their visual and auditory systems in particular have been the subject of intensive research (Wylie et al., 2015; Iwaniuk and Wylie, 2020). For instance, DRD4 is present in the retina (Macisaac et al., 2024; Klitten et al., 2008), where it contributes to light adaptation (Flood and Eggers, 2021). The olfactory system in birds is important not only for food location but also for navigation in homing pigeons (Gagliardo and Bingman, 2024), nest recognition, predator avoidance, reproductive control, including social interaction (Balthazart and Taziaux, 2009), and behavioral ecology (Corfield et al., 2015). There is evidence that birds such as seabirds (Nevitt et al., 1995), penguins (Amo et al., 2013), and turkey vultures (Smith et al., 2002; Grigg et al., 2017) use their sense of olfaction in their foraging behavior. In this study, we revealed that the A16 DA neuron population is present in the GrO, which is rich in GABAergic interneurons, in chicks, similar to the cellular composition of the mammalian OB. We also found that *DRD*4, a member of the D2 family, is expressed in chick mitral cells, similar to that in mammalian mitral cells. These findings suggest that the neural circuits in the OB that process olfactory information are likely to be well-conserved between birds and mammals. Physiological confirmation using avian OB is required in the future.

We also examined the expression patterns of the other *DRD*s in the chick forebrain; however, no signal was detected for *DRD1E*. The expression patterns of *DRD1, DRD2, DRD5*, and *DRD1C* were consistent with those reported in previous studies (Kubikova et al., 2010; Yamamoto et al., 2013), while previously unknown expression regions were identified for *DRD3* and *DRD4*. Specifically, *DRD3* was observed in the IHA, entopallium, and V-shape region in the HF, suggesting that DA modulation *via* DRD3 may play an important role in these regions. We previously revealed that in the avian HF, different subtypes of receptors for serotonin, a different monoamine neuromodulator, are expressed in different populations in each subregion (Fujita et al., 2020, 2022a) and that they are expressed in serotonergic neurons (Fujita et al., 2022b). A very small population expressing *5-HTR1A, 5-HTR1B*, and *5-HTR3A* was observed in the V-shape region, indicating that serotonergic modulation of the avian HF is characteristic and important (Fujita et al., 2023). Previous studies have established that *DRD5* and *DRD1C*, both D1 family receptors, are expressed in the V-shape region (Kubikova et al., 2010; Yamamoto et al., 2013). In contrast, our study identifies the expression of *DRD3*, a D2 family receptor, further complicating the understanding of dopamine modulation in this region. Therefore, both D1 and D2 receptors are widely expressed in the V-shape region of birds, suggesting that dopamine, in conjunction with serotonin, plays a pivotal role in the functional regulation of this area.

In conclusion, we comprehensively described the expression distribution of DA neuron-related markers in the chick forebrain, revealing that monoenzymatic DA neurons are also distributed in the avian brain. These findings suggest that the presence of monoenzymatic DA neurons may represent a conserved feature of the vertebrate DA system. Furthermore, we found that *DRD4*, an avian personality gene, is highly selectively expressed in the mitral cells of the OB and revealed expression sites for other DRDs, including previously undescribed sites. Our findings will enhance our understanding of DA regulation in the avian forebrain and provide insight into how the personality gene *DRD4* contributes to the regulation of brain functions in the avian brain.

## Data Availability

The raw data supporting the conclusions of this article will be made available by the authors, without undue reservation.
