# Supplementary material for: Molecular characterization of chicken DA systems reveals that the avian personality gene, DRD4, is expressed in the mitral cells of the olfactory bulb
Source: Front Neuroanat. 2025 Jan 15;19:1531200. doi: 10.3389/fnana.2025.1531200 (PMC11774857; doi:10.3389/fnana.2025.1531200)
Supplement: Supplementary file 1 [file Table_1.pdf]

Supplemental Table 1. Subject summary used in this study

	probes										
	TH	***DDC***	***DBH***	***DRD1***	***DRD2***	***DRD3***	***DRD4***	***DRD5***	***DRD1C***	***DRD1E***	***GAD2***
chick #s											
**#1**	○	○	○	○	○	○	○	○	-	-	-
**#2**	○	○	○	-	○	○	-	-	○	○	-
**#3**	○	○	○	○	○	○	○	○	○	○	-
**#4**	○	○	○	-	○	○	○	○	○	○	-
**#5**	○	○	○	-	○	○	○	-	○	-	○
**#6**	○	○	○	-	-	○	○	○	○	-	-
**#7**	○	○	○	-	-	-	-	-	-	-	-
**#8**	○	○	○	○	-	-	-	○	○	-	-
**#9**	○	○	○	-	-	-	-	-	-	-	-
**#10**	○	○	○	-	-	-	-	-	-	-	-
**#11**	○	○	○	-	-	-	-	-	-	-	-
**#12**	○	○	○	-	-	-	○	-	-	-	○
**#13**	○	-	-	-	-	-	○	-	-	-	○
**#14**	○	-	-	-	○	○	○	-	-	-	○
**#15**	○	-	-	-	○	○	○	-	-	○	○
**#16**	-	-	-	○	○	-	○	○	-	○	-
**#17**	-	-	-	○	○	-	○	○	-	-	-
**#18**	-	-	-	○	○	-	-	○	○	○	-
**#19**	-	-	-	○	-	-	-	○	-	-	-
**#20**	-	-	-	○	-	○	-	-	○	○	-
**#21**	-	-	-	○	-	○	○	-	-	○	-
**#22**	-	-	-	-	○	○	-	-	○	○	-

Supplemental Table 2. Summary of sequence comparison of chicken DRDs used in this study

		**Identity(%)**	
**Chicken gene**	**Opponent**	**DNA**	**Protein**
DRD1	*H. sapiens*	77.5	82.7
DRD2	*H. sapiens*	78.7	81.2
DRD3	*H. sapiens*	70.9	71.3
DRD4	*H. sapiens*	66.4	61
DRD5	*H. sapiens*	75.7	77.6
DRD1C	*D. rerio*	65.4	62.2
DRD1E	*D. rerio*	55	63
