# Supplementary figures and images for: Molecular characterization of chicken DA systems reveals that the avian personality gene, DRD4, is expressed in the mitral cells of the olfactory bulb
Source: Front Neuroanat. 2025 Jan 15;19:1531200. doi: 10.3389/fnana.2025.1531200 (PMC11774857; doi:10.3389/fnana.2025.1531200)

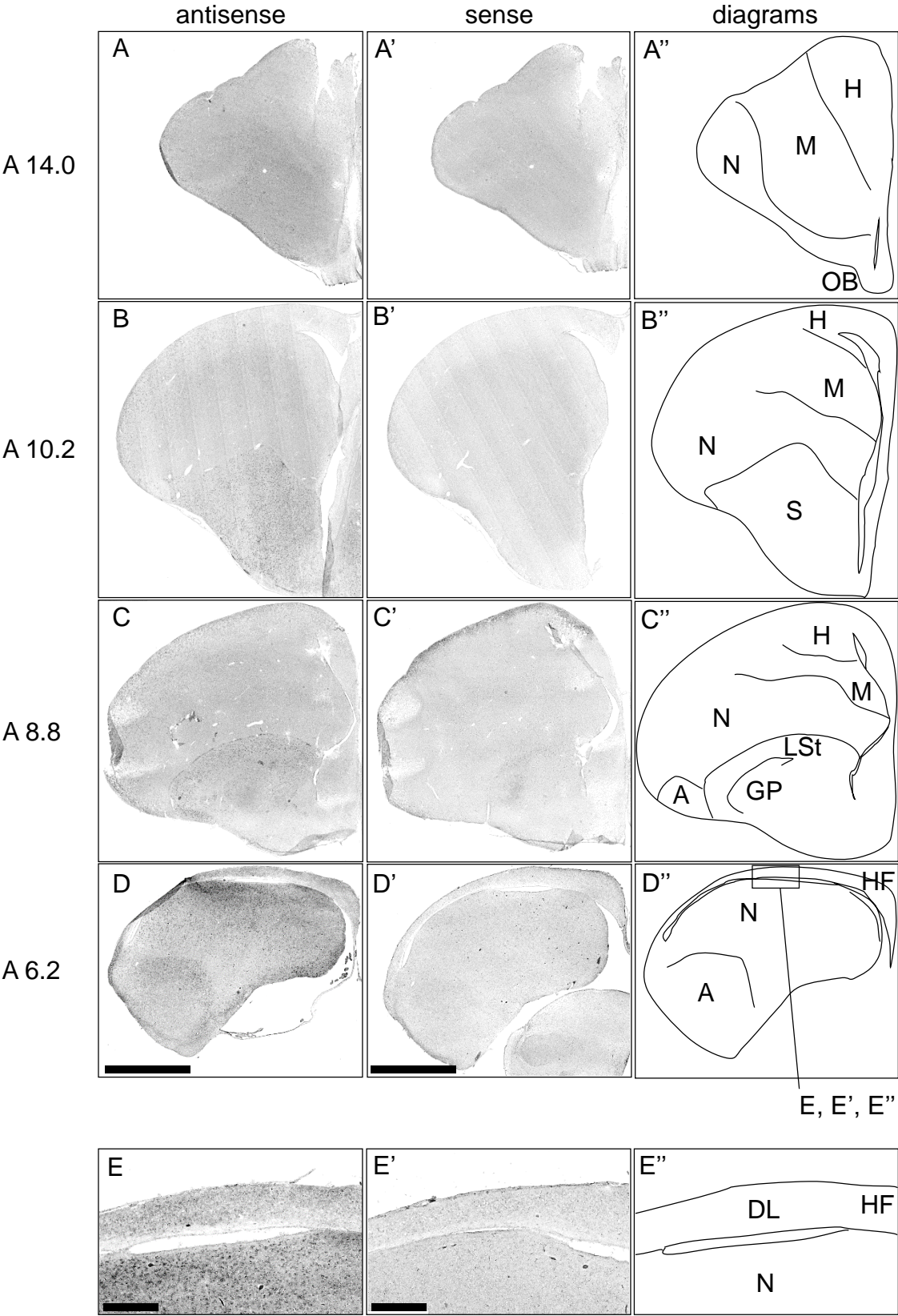

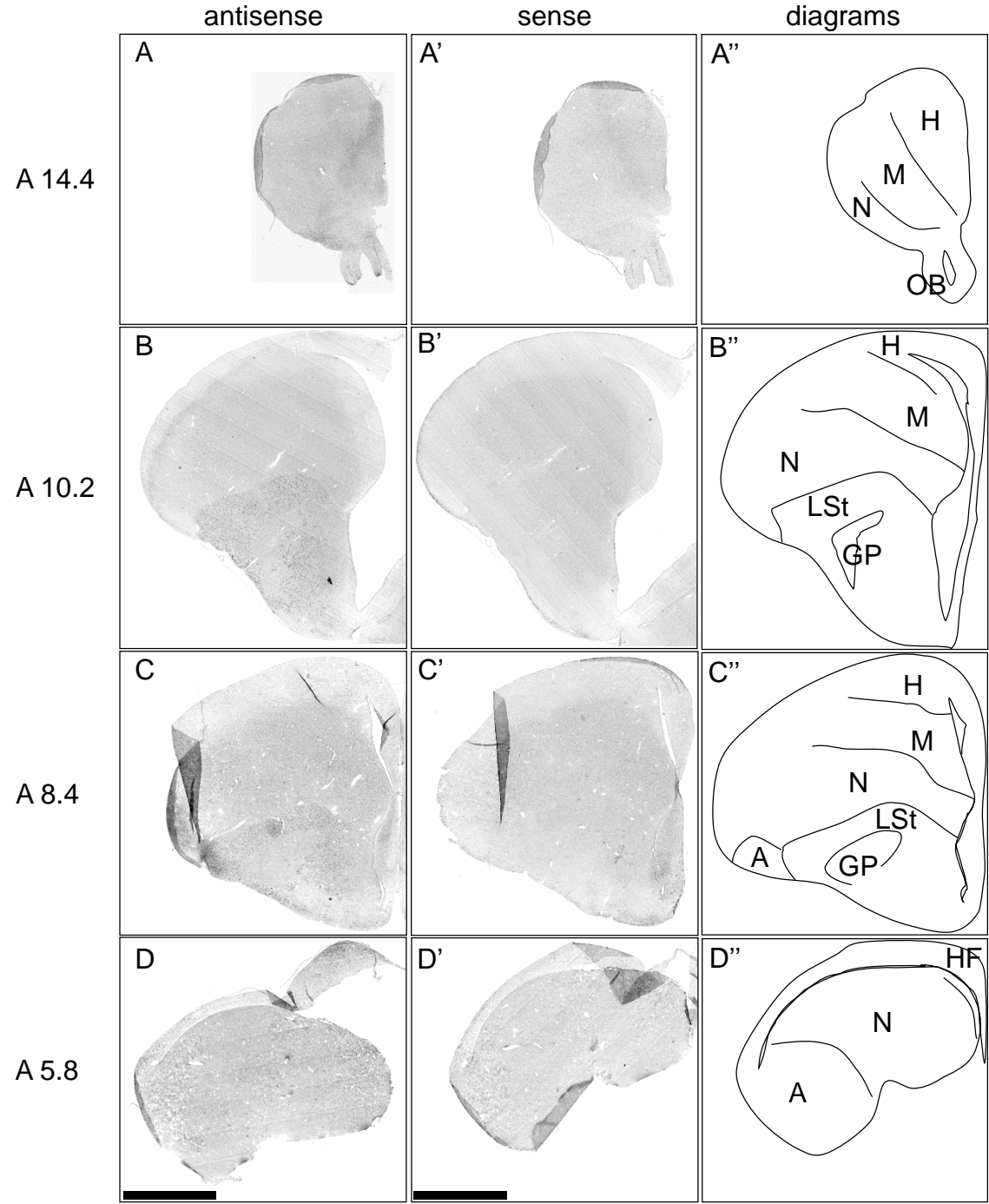

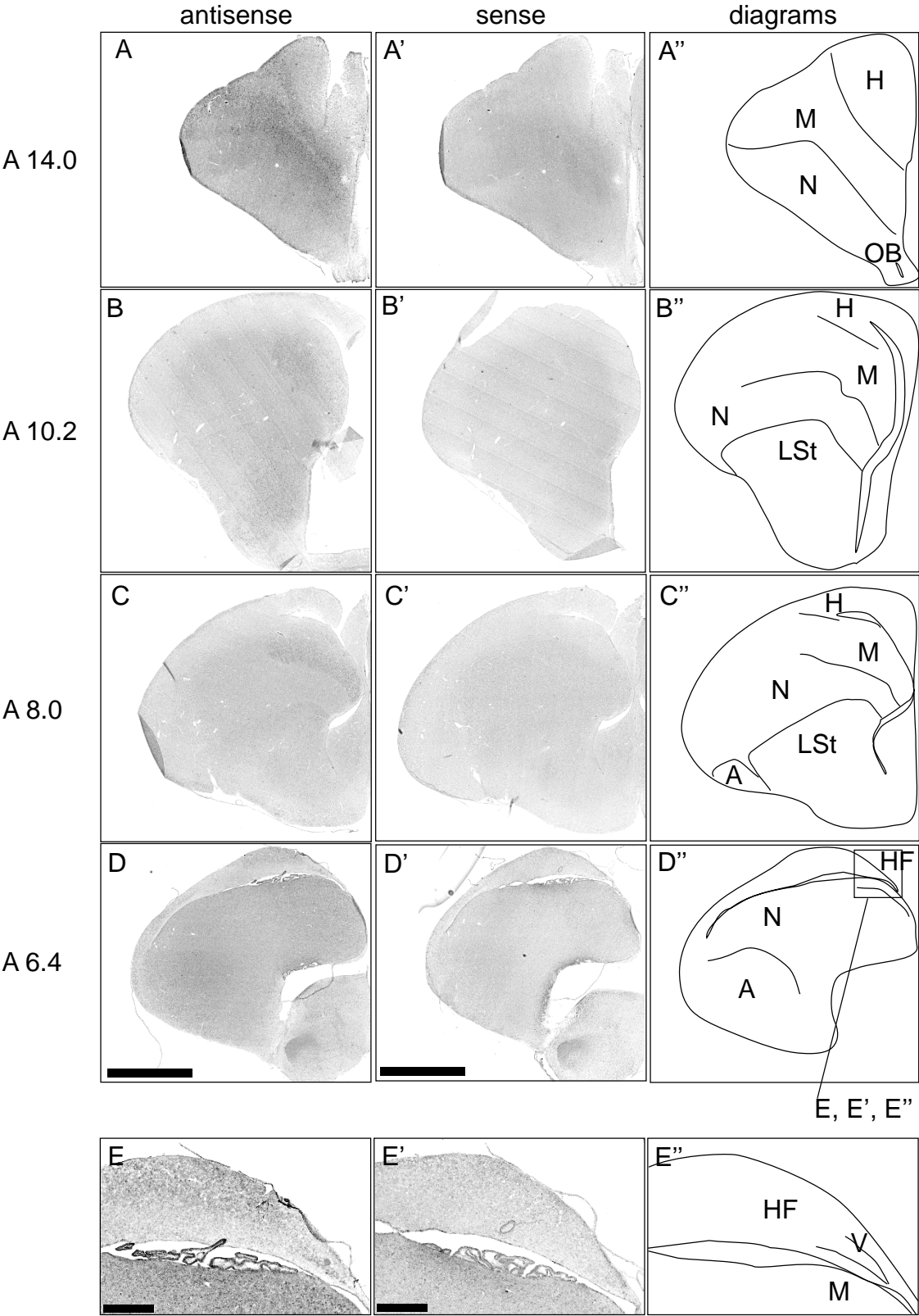

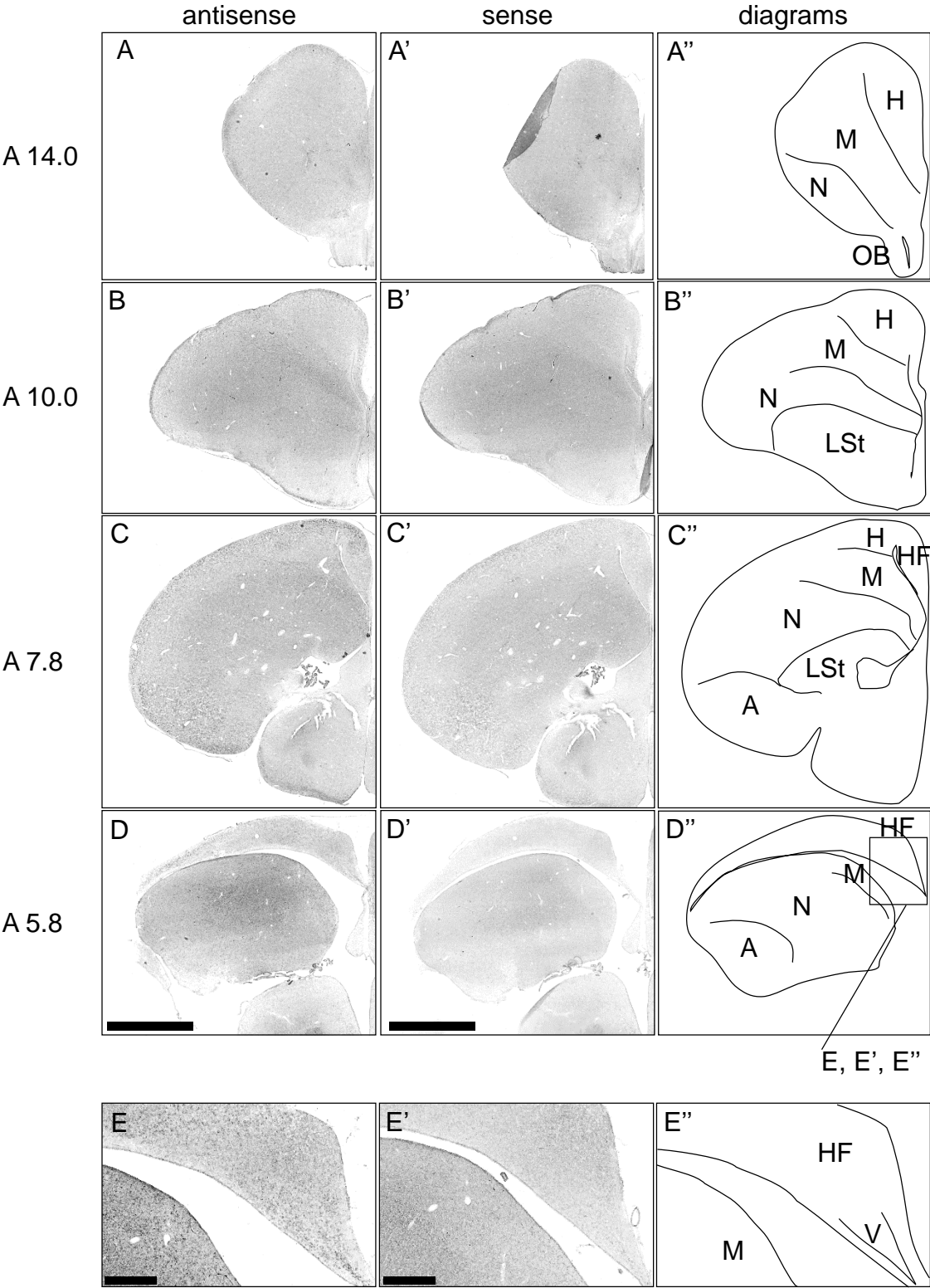

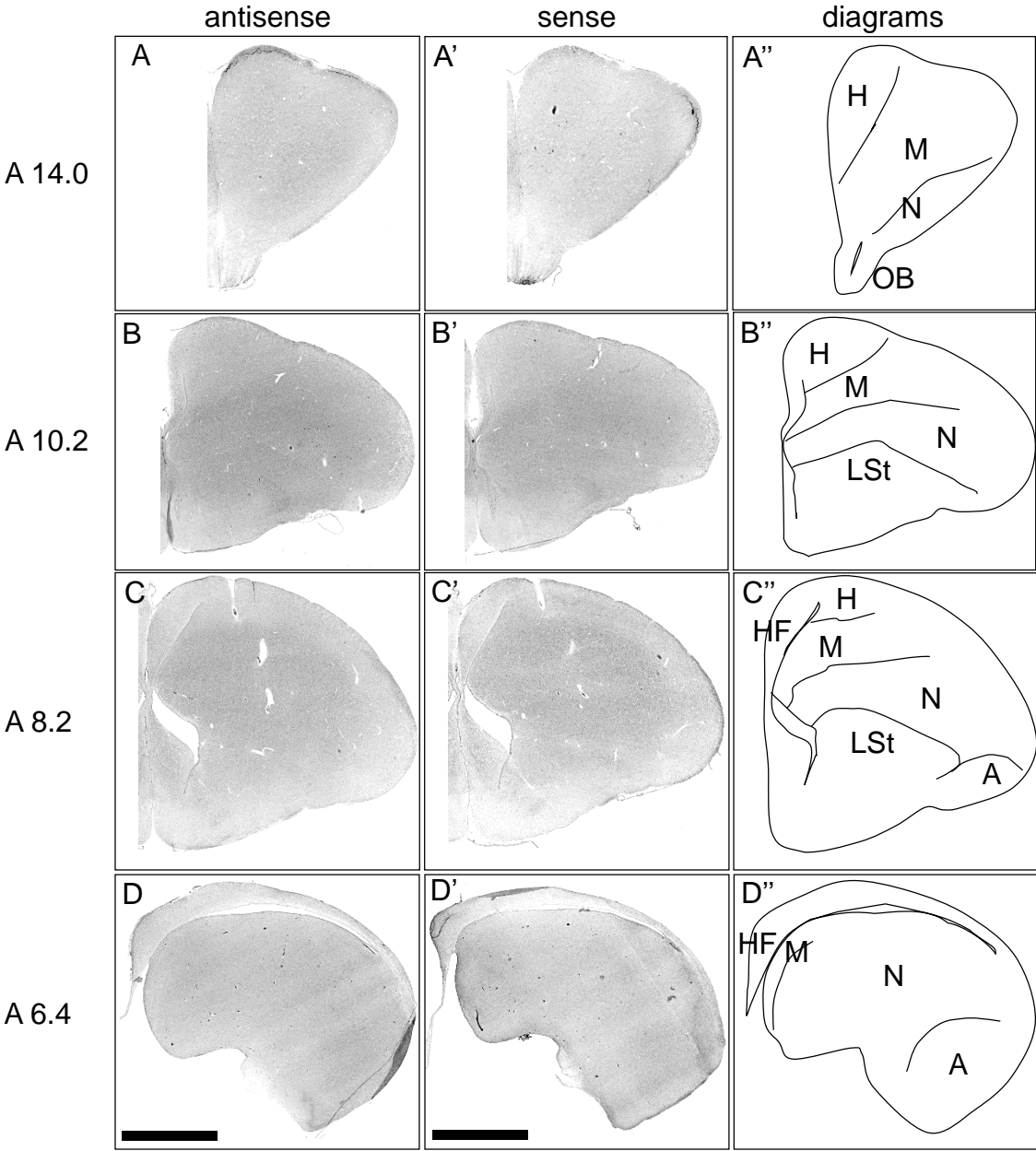

Supplement: Supplementary file 2 [file Presentation_1.pdf]
