# Supplementary material for: Molecular characterization of chicken DA systems reveals that the avian personality gene, DRD4, is expressed in the mitral cells of the olfactory bulb
Source: Front Neuroanat. 2025 Jan 15;19:1531200. doi: 10.3389/fnana.2025.1531200 (PMC11774857; doi:10.3389/fnana.2025.1531200)
Supplement: Supplementary file 3 [file Presentation_2.pdf]

Supplemental figure legends

Supplemental Figure 1. *In situ* hybridization of *DRD1* in P1 chick telencephalons

Digoxigenin-labelled RNA probes, including antisense (**A–E**) and sense (**A’–E’**) *DRD1* probes, were used for *in situ* hybridization in coronal sections of the P1 chick telencephalon. Signal reproducibility was verified across multiple chicks at the same level, with representative images provided. Diagrams of coronal sections are shown in the rightmost panels (**A’’–E’’**). Levels of sections (A 14.0 to A 6.2) were in accordance with those mentioned in Kuenzel and Masson’s chick atlas (Kuenzel and Masson, 1988). Panels (**E** and **E’**) show magnified views of the HF region indicated by the box in (**D’’**). A: arcopallium; DL: dorsolateral region; G: globus pallidus; H: hyperpallium; HF: hippocampal formation; LSt lateral striatum; M, mesopallium; N, nidopallium; OB: olfactory bulb; S: striatum. Scale bars = 2.5 mm (**A-D** and **A’-D’**) and 500 µm (**E** and **E’**).

Supplemental Figure 2. *In situ* hybridization of *DRD2* in P1 chick telencephalons

Digoxigenin-labelled RNA probes, including antisense (**A–D**) and sense (**A’–D’**) *DRD2* probes, were used for *in situ* hybridization in coronal sections of the P1 chick telencephalon. Signal reproducibility was verified across multiple chicks at the same level, with representative images provided. Diagrams of coronal sections are shown in the rightmost panels (**A**–**D’’**). Levels of sections (A 14.4 to A 5.8) were in accordance with those mentioned in Kuenzel and Masson’s chick atlas (Kuenzel and Masson, 1988). A: arcopallium; G: globus pallidus; H: hyperpallium; HF: hippocampal formation; LSt: lateral striatum; M: mesopallium; N: nidopallium; OB: olfactory bulb. Scale bars = 2.5 mm.

Supplemental Figure 3. *In situ* hybridization of *DRD5* in P1 chick telencephalons

Digoxigenin-labelled RNA probes, both antisense (**A–E**) and sense (**A’–E’**) *DRD5,* were used for *in situ* hybridization in coronal sections of the P1 chick telencephalon. Signal reproducibility was verified across multiple chicks at the same level, with representative images provided. Diagrams of coronal sections are shown in the rightmost panels (**A’’–E’’**). Levels of sections (A 14.0 to A 6.4) were in accordance with those mentioned in Kuenzel and Masson’s chick atlas (Kuenzel and Masson, 1988). Panels (**E** and **E’**) show magnified views of the HF region indicated by the box in (**D’’**). A: arcopallium; H: hyperpallium; HF: hippocampal formation; LSt: lateral striatum; M: mesopallium; N: nidopallium; OB: olfactory bulb; V: V-shape region. Scale bars = 2.5 mm (**A-D** and **A’-D’**) and 500 µm (**E** and **E’**).

Supplemental Figure 4. ***In situ*** **hybridization of** ***DRD1C*** **in P1 chick telencephalons**

Digoxigenin-labelled RNA probes, both antisense (**A–E**) and sense (**A'–E'**) *DRD1C*, were used for *in situ* hybridization in coronal sections of the P1 chick telencephalon. Signal reproducibility was verified across multiple chicks at the same level, with representative images provided. Diagrams of coronal sections are shown in the rightmost panels (**A''–E''**). Levels of sections (A 14.0 to A 5.8) were in accordance with those mentioned in Kuenzel and Masson's chick atlas (Kuenzel and Masson, 1988). Panels (**E** and **E'**) show magnified views of the HF region indicated by the box in (**D''**). A: arcopallium; G: globus pallidus; H: hyperpallium; HF: hippocampal formation; LSt: lateral striatum; M: mesopallium; N: nidopallium; OB: olfactory bulb; V: V-shape region. Scale bars = 2.5 mm (**A-D** and **A'-D'**) and 500 µm (**E** and **E'**).

Supplemental Figure 5. ***In situ*** **hybridization of** ***DRD1E*** **in P1 chick telencephalons**

Digoxigenin-labelled RNA probes, both antisense (**A–D**) and sense (**A'–D'**) *DRD1E*, were used for *in situ* hybridization in coronal sections of the P1 chick telencephalon. Signal reproducibility was verified across multiple chicks at the same level, with representative images provided. Diagrams of coronal sections are shown in the rightmost panels (**A**–**D''**). Levels of sections (A 14.0 to A 6.4) were in accordance with those mentioned in Kuenzel and Masson's chick atlas (Kuenzel and Masson, 1988). A: arcopallium; H: hyperpallium; HF: hippocampal formation; LSt: lateral striatum; M: mesopallium; N: nidopallium; OB: olfactory bulb. Scale bars = 2.5 mm.
